# Genetic Bases of the Stomata-Related Traits Revealed by a Genome-Wide Association Analysis in Rice (*Oryza sativa* L.)

**DOI:** 10.3389/fgene.2020.00611

**Published:** 2020-06-09

**Authors:** Hongwei Chen, Xiuqin Zhao, Laiyuan Zhai, Kuitian Shao, Kunwei Jiang, Congcong Shen, Kai Chen, Shu Wang, Yun Wang, Jianlong Xu

**Affiliations:** ^1^Rice Research Institute, Shenyang Agricultural University, Shenyang, China; ^2^Institute of Crop Sciences/National Key Facility for Crop Gene Resources and Genetic Improvement, Chinese Academy of Agricultural Sciences, Beijing, China; ^3^Shenzhen Branch, Guangdong Laboratory for Lingnan Modern Agriculture, Genome Analysis Laboratory of the Ministry of Agriculture, Agricultural Genomics Institute at Shenzhen, Chinese Academy of Agricultural Sciences, Shenzhen, China

**Keywords:** stomatal density, stomatal size, genome-wide association study, QTL, candidate gene

## Abstract

Stomatal density (*D*) and size (*S*) are an important adaptive mechanism for abiotic stress tolerance and photosynthesis capacity in rice. However, the genetic base of rice stomata-related traits still remains unclear. We identified quantitative trait loci (QTLs) associated with *D* and *S* on abaxial and adaxial leaf surfaces using genome-wide association analysis with 451 diverse accessions in two environments. *D* and S showed significant differences between *indica* (*xian*) and *japonica* (*geng*) accessions and significantly negative phenotypic correlations. A total of 64 QTLs influencing eight stomata-related traits were identified using 2,936,762 high-quality single nucleotide polymorphism markers. Twelve QTLs were consistently detected for the same traits in nine chromosomal regions in both environments. In addition, 12 QTL clusters were simultaneously detected for the same stomata-related traits on abaxial and adaxial leaf surfaces in the same environment, probably explaining the genetic bases of significant correlations of the stomata-related traits. We screened 64 candidate genes for the nine consistent QTL regions using haplotype analysis. Among them, *LOC_Os01g66120* for *qD_*ada*_1*, *OsSPCH2* (*LOC_Os02g15760*) for *qD_*ada*_2.1* and *qD_*aba*_2.1*, *LOC_Os02g34320* for *qS_*ada*_2.2*, *OsFLP* (*LOC_Os07g43420*) or *LOC_Os07g43530* for *qS_*aba*_7.1*, and *LOC_Os07g41200* for *qW_*ada*_7* and *qW_*aba*_7* were considered as the most likely candidate genes based on functional annotations. The results systematically dissected the genetic base of stomata-related traits and provide useful information for improving rice yield potential via increasing abiotic stress tolerance and photosynthesis capacity under stressed and non-stressed conditions through deploying the favorable alleles underlying stomata-related traits by marker-assisted selection.

## Introduction

Plant stomata are microscopic pores on the epidermis of leaves formed by a pair of specialized guard cells, and some are surrounded by subsidiary cells in certain monocot species such as rice (Bergmann and Sack, 2007). Stomata act as a gateway for the efficient exchange of water vapor and CO_2_ between plants and the atmosphere. Stomatal size (*S*) and density (*D*) are two major factors that improve stomatal conductance and photosynthetic capacity, which are dramatically associated with the grain yield of crops under stressed and non-stressed conditions (Xu and Zhou, 2008; Franks and Beerling, 2009). Plant stomatal density plays an important role in plant responses to environmental conditions, such as elevated CO_2_ concentration (Xu et al., 2016), salt stress (Wei et al., 2019), drought stress (Zandalinas et al., 2018), and light intensity (Shimazaki et al., 2007). Moreover, many studies have reported that water deficit leads to an increase in *D*, and a decrease in *S* (Xu and Zhou, 2008; Buckley et al., 2020), indicating that this might be partially related to enhancing the adaptation of plants to drought.

Differences in *D* were observed between the abaxial (lower) and adaxial (upper) leaf surfaces. More specifically, the abaxial surface possesses more stomata compared with the adaxial surface (Ishimaru et al., 2001; Laza et al., 2010), and significant positive correlations were identified for stomatal density between both sides (Ishimaru et al., 2001). Generally, *xian* rice varieties tend to have higher *D* and smaller *S* than *geng* varieties (Laza et al., 2010). Significant negative correlations were also observed between *D* and *S* in rice (Ishimaru et al., 2001; Ohsumi et al., 2007; Laza et al., 2010). However, the genetic basis of *D* and *S* on adaxial and abaxial leaf surfaces is still poorly understood. Therefore, it is important to study the genetic basis of stomata-related traits to provide useful information for the genetic improvement of rice to enhance rice photosynthetic abilities and stress tolerance.

The use of quantitative trait locus (QTL) mapping has contributed to a better understanding of the genetic basis of many agronomically important traits. Over the last few decades, many researchers have identified QTLs for stomata-related traits, including *D* and *S*, using bi-parental populations in rice (Ishimaru et al., 2001; Laza et al., 2010). Notably, several genes controlling stomatal development in *Arabidopsis*, such as *TOO MANY MOUTHS* (*TMM*) (Geisler et al., 2000), *STOMATAL DENSITY AND DISTRIBUTION 1* (*SDD1*) (Von Groll et al., 2002), *MITOGEN-ACTIVATED PROTEIN KINASE3* (*MPK3*) and *MPK6* (Wang et al., 2007), *STOMAGEN* (Kondo et al., 2010), which encode putative receptors, proteases, or kinases, and act primarily by modulating the number and placement of asymmetric divisions in the stomatal cell lineage, respectively. In rice, certain key transcriptional factors (TFs), such as *SPEECHLESS* (*OsSPCH*), *MUTE* (*OsMUTE*), *FAMA* (*OsFAMA*), *INDUCER OF CBF EXPRESSION1* (*OsICE*), *FOUR LIPS* (*OsFLP*) regulate the stomatal development stage (Wu et al., 2019). Mohammed et al. (2019) reported overexpressed rice *EPIDERMAL PATTERNING FACTOR1* (*OsEPF1*) produced transgenic plants with reduced stomatal densities, resulting in decreased leaf stomatal conductance and enhanced water use efficiency.

A genome-wide association study (GWAS) is an effective method to dissect the genetic basis of complex traits, such as grain yield, and biotic and abiotic stress tolerances (Zhao et al., 2018; Zhai et al., 2018; Liu et al., 2019). In the present study, we reported the first GWAS to identify QTLs for stomata-related traits in rice using a panel of germplasm resources with a broad genetic diversity. A diverse panel consisting of 451 accessions from the 3000 Rice Genomes Project (3K RGP) (3K RGP, 2014) was used to detect QTLs for stomata-related traits in rice and identify associated candidate genes by GWAS using 4.8M single nucleotide polymorphisms (SNPs) generated using high-throughput re-sequencing. The results will shed new light on the genetic basis of stomata-related traits and provide superior alleles to improve rice stomata-related traits by molecular breeding.

## Materials and Methods

### Plant Materials

The 451 worldwide accessions, which originated from 60 countries, were collected from 3K RGP (3K RGP, 2014). Based on the known population structure and division of subpopulations (Wang et al., 2018), the panel contained 271 *xian* accessions (66 *xian*-1A, 75 *xian*-1B, 20 *xian*-2, 1 *xian*-3, and 109 *xian*-adm), 145 *geng* accessions (63 *geng* -tmp, 6 *geng*-sbtrp, 53 *geng*-trp, and 23 *geng*-adm), 11 aus/boro, 5 basmati/sadri and 19 highly admixture (*adm*) accessions (Supplementary Table S1).

### Phenotypic Investigation

All of these genotypes were grown in Sanya (SY, 18.3° N, 109.3° E) during December 2016–April 2017 and Beijing (BJ, 40.2° N, 116.2° E) during May–October, 2017, and each accession was planted in two rows with 10 individuals in each row at a spacing of 25 cm × 17 cm for two replications. Field management followed the local standard management practices. To minimize flowering time effects on possible experiment error, batch samplings were conducted based on the heading date of each accession. At the full heading stage, eight uniform flag leaves from the main stem of eight plants (one main stem from one plant) in the middle of each plot were collected and kept in formalin-acetic-alcohol (FAA) fixative solution. Leaf impressions were made on the adaxial and abaxial leaf surfaces at the middle part of the flag leaf. The stomatal density (stomata per unit area) on the adaxial leaf surface (*D*_ada_, number/mm^2^) and abaxial leaf surface (*D*_aba_, number/mm^2^) was counted at 500 × magnification and repeated in five impressions from each of leaf using a scanning electron microscope (Hitachi TM3030, Tokyo, Japan). The guard cell length (*L*) on the adaxial leaf surface (*L*_ada_, μm) and abaxial leaf surface (*L*_aba_, μm), and guard cell width (*W*) on the adaxial leaf surface (*W*_ada_, μm) and abaxial leaf surface (*W*_aba_, μm), were observed and measured at 1200 × magnification and repeated for five replicates. The *S* values on the adaxial leaf surface (*S*_ada_, μm^2^) and abaxial leaf surface (*S*_aba_, μm^2^) were calculated based on the assumption that stomata are elliptical in shape, with their major axis equal to *L* and their minor axis equal to *W*, as follows (Xiong et al., 2018; Zhang et al., 2019):

$$S=\frac{L}{2}\times \frac{W}{2}\times \pi$$

The average trait value of each accession was used for data analyses.

### Genotyping

The 4.8M SNP genotype data of this association panel were selected from the 3K RGP (3K RGP, 2014). SNPs with a missing rate over 20% and a minor allele frequency less than 5% were removed. Heterozygous alleles were also eliminated. Finally, a total of 2,936,762 high quality SNP markers that were evenly distributed on chromosomes were used to carry out the GWAS.

### QTL Mapping by GWAS

We performed a GWAS to detect SNPs that were significantly associated with all stomata-related traits using 2,936,762 SNPs and the mean trait values of the 451 accessions from each of the environments. Principal component analysis (PCA) was conducted using the efficient mixed-model association (EMMA) method in the Genome Association and Prediction Integrated Tool (GAPIT) R package (Lipka et al., 2012) to examine the population structure. The K matrix (kinship matrix) was calculated by the EMMAX software based on the Bayesian Network (BN) method using those high quality SNPs. Marker-trait associations were conducted by the model of mixed linear (MLM), PCA+K, implemented in GAPIT. The critical *P*-value for declaring significant marker-trait association (1.0 × 10^–5^) was calculated using GEC software based on the independent effective SNP number (Li et al., 2012).

We realized that the use of a single *P*-value threshold in GWAS could result in detection of a significant marker-trait associated SNP in one environment but not in another environment. To further examine the extent to which inconsistent associated SNP detection across the two environments (SY and BJ) actually occurred from type II errors, all significant associated SNPs identified under one condition were re-examined using the data from the other condition, with a minimum significance threshold of *P* < 1.0 × 10^–4.5^. The test statistics and QTL parameters associated with the QTLs were also reported as long as the QTLs reached the minimum threshold (Xu et al., 2005; Li, 2001). To estimate independently associated regions of identified QTLs, significantly trait-associated SNPs situated in one estimated LD block were defined as the same QTL. Each LD block containing the identified SNPs was evaluated with the R package “LDheatmap” (Hyung et al., 2006) according to the block definition suggested by Yano et al. (2016).

### Identification of Candidate Genes

The haplotypes of all the genes located in the candidate regions for QTLs consistently detected in the two environments from the Rice Annotation Project Database^1^ (Sakai et al., 2013) were conducted according to all available non-synonymous SNPs located inside of these genes in the 451 rice accessions. Haplotypes containing more than 10 rice accessions were used to analyze the significant differences in phenotype. Six representative candidate genes were selected for a comprehensive analysis based on the significance of the haplotype analyses [analysis of variance (ANOVA)], their biochemically related functions, and their expression profiles. Gene expression profiles were downloaded from a rice expression profile database [RiceXPro (version 3.0)]^2^ (Sato et al., 2013). Two-sided Fisher’s exact tests in R were used to compare haplotype frequencies between *xian* and *geng* subpopulations.

### Statistical Analysis

Differences in the mean phenotypic values among the haplotypes (containing more than 10 accessions) were evaluated using a one-way ANOVA. Duncan’s multiple mean comparison test was carried out to determine the significance of any differences (5% significance level) using the agricolae package in R. Phenotypic correlation analyses of the eight stomata-traits were computed using the corrplot package in R. Variance components were evaluated using multiple-site analysis with all effects treated as random. We computed heritability across the two environments using the estimated variance components as *V*_*G*_/(*V*_*G*_ + *V*_*GEI*_/*s* +*V*_*e*_/*sr*), where *V*_*G*_, *V*_*GEI*_, and *V*_*e*_ are the variances of genotype, genotype-by-environment interaction variance, and residual error, respectively; *s* is the number of environments; and *r* is the number of replicates. All analyses were conducted with the PBTools package^3^ developed by The International Rice Research Institute.

## Reults

### Phenotypic Variation of Stomata-Related Traits

The rice panel used in this study showed wide variations for all the investigated traits and most traits appeared to be normally distributed (Supplementary Table S1 and Supplementary Figure S1). The 451 accessions presented substantial variations for the tested stomata-related traits in Sanya (SY) and Beijing (BJ). Comparing the *D* and *S* values on the abaxial leaf surface with those on the adaxial leaf surface revealed that *D*_aba_ (average 739.7 number/mm^2^ and 683 number/mm^2^ in SY and BJ, respectively) was significantly higher than *D*_ada_ (average 548.2 number/mm^2^ and 517.6 number/mm^2^ in SY and BJ, respectively) (Supplementary Figure S2). In addition, *S*_aba_ was significantly larger than *S*_ada_ in BJ, whereas no significant difference was observed between the two sides in SY (Supplementary Figure S2). Based on estimates of variance components for the eight traits, most traits were controlled mainly by *V*_*G*_, whereas *V*_*GEI*_ was the main source for *L*_ada_ and *S*_ada_ (Supplementary Table S2). The heritability of the eight traits ranged from 0.41 for *L*_ada_ to 0.69 for *D*_ada_ (Supplementary Table S2).

**FIGURE 1:**
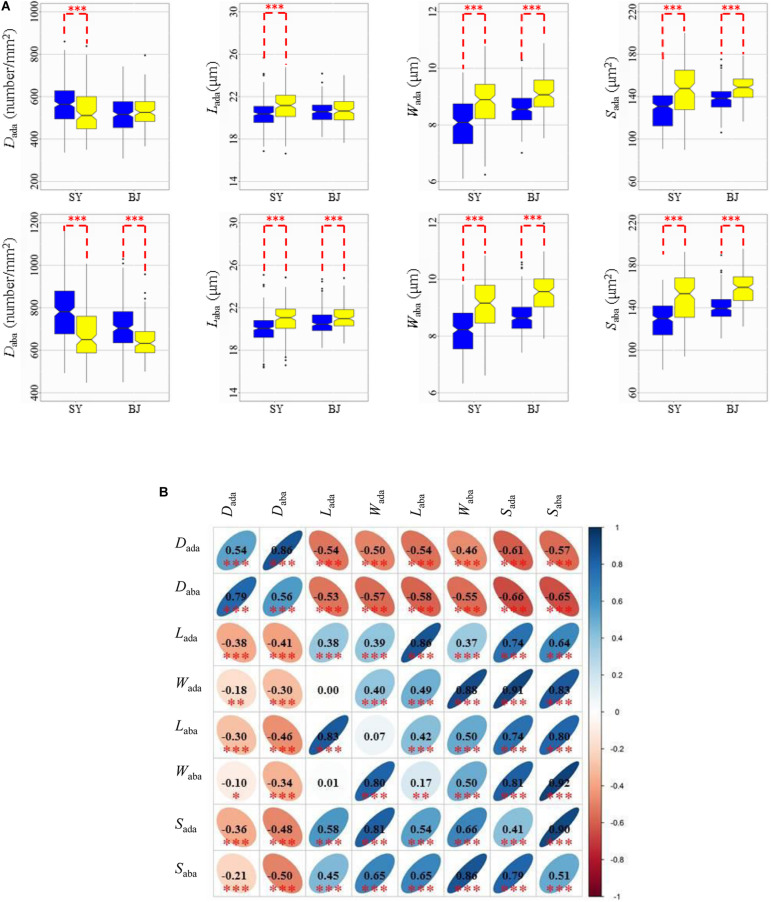
Values and correlations of stomata-related traits in two environments. **(A)** Box plots of eight stomata-related traits in Sanya (SY) and Beijing (BJ) between *xian* and *geng* rice (*Oryza sativa* L.) panels. Blue and yellow colors indicate *xian* and *geng*, respectively. ***Denotes significance of Student’s *t*-test at *P* < 0.001. *D*_ada_, stomatal density on the adaxial surface; *D*_aba_, stomatal density on the abaxial surface; *L*_ada_, guard cell length on the adaxial surface; *L*_aba_, guard cell length on the abaxial surface; *W*_ada_, guard cell width on the adaxial surface; *W*_aba_, guard cell width on the abaxial surface; *S*_ada_, stomatal size on the adaxial surface; *S*_aba_, stomatal size on the abaxial surface. **(B)** Correlations between the eight tested traits in SY (upper diagonal) and BJ (lower diagonal). The values are correlation coefficients (*r*) multiplied by 100. The values on the main diagonal are correlations between SY and BJ. The areas and colors of ellipses correspond to absolute values of the corresponding *r*. Right and left oblique ellipses indicate positive and negative correlations, respectively. Values without glyphs were insignificant at the 0.05 probability level. *, **, *** Indicate significant correlations at *P* < 0.05, *P* < 0.01, and *P* < 0.001, respectively.

Based on the results from 3,010 rice accessions (Wang et al., 2018), 271 accessions were classified into the *xian* subpopulation, and 145 accessions were classified into the *geng* subpopulation for further phenotypic analysis in the two subpopulations. *Xian* varieties exhibited significantly higher *D*, but significantly smaller *L*, *W*, and *S* than *geng* varieties on the two sides in the two environments, except *D*_ada_ and *L*_ada_ in BJ (Figure 1A).

In the two environments, the trends of pairwise phenotypic correlations were similar. Phenotypic correlations analysis demonstrated that significantly positive correlations of *D* and *S* could be observed between the abaxial and adaxial leaf surfaces. The *D* value had strong negative correlations with *S* for the abaxial and adaxial leaf surfaces. As expected, *S* showed significant positive correlations with its corresponding component traits (*L* and *W*) on the two sides of the leaves in the two environments (Figure 1B).

### Basic Statistics of Markers

A total of 2,936,762 high density SNPs with minor allele frequencies greater than 5% and missing rates of less than 20% were used in this study. The number of markers per chromosome varied from 168,424 on chromosome 9 to 359,991 on chromosome 1. The size of the chromosomes ranged from 22.9 Mb for chromosome 9 to 43.3 Mb for chromosome 1. The whole genome size was 372.9 Mb, with an average marker spacing of 127.8 bp, which ranged from 108.8 bp on chromosome 10 to 152.2 bp on chromosome 5 (Supplementary Table S3).

### Identification of QTLs for Stomata-Related Traits by GWAS

Total of 723 and 570 significant SNPs associated with stomata-related traits on the adaxial and abaxial surfaces were identified on 12 chromosomes, respectively, in SY and BJ (Table 1 and Supplementary Figure S3). Adjacent significantly associated SNPs located in one estimated linkage disequilibrium (LD) block were defined as one QTL. Thus, a total of 64 QTLs were identified for the eight traits in the two environments (Table 1), including 34 QTLs detected only in SY, 18 detected only in BJ, and 12 detected simultaneously in both environments, including five (*qD_*ada*_1*, *qD_*ada*_2.1*, *qD_*ada*_2.2*, *qD_*ada*_4*, and *qD_*ada*_6*), one (*qW_*ada*_7*), two (*qS_*ada*_2.2* and *qS_*ada*_4.2*), one (*qD_*aba*_2.1*), one (*qW*_aba_*7*), and two (*qS_*aba*_4.2* and *qS_*aba*_7.1*) for *D*_ada_, *W*_ada_, *S*_ada_, *D*_aba_, *W*_aba_, and *S*_aba_, respectively.

**TABLE 1:** QTLs identified for stomata-related traits by GWAS in two environments.

**Trait**	**QTL**	**Chr**	**Envir.**	**No. of significant SNPs**	**Peak SNP**	**Interval (Mb)**	***P*-value of peak SNP**	**Previously reported QTLs and genes**
*D*_ada_	*qD_*ada*_1*	1	SY	20	rs1_38402831	38.32–38.63	1.4E-06	*OsNAC6* (Nakashima et al., 2007)
			BJ	1	rs1_38402831	38.32–38.63	3.1E-05	
	*qD_*ada*_2.1*	2	SY	15	rs2_8970096	8.87–9.05	4.5E-06	*OsSPCH2* (Liu et al., 2009);
			BJ	138	rs2_9038842	8.87–9.05	1E-07	QTL for △^13^C (Takai et al., 2009)
	*qD_*ada*_2.2*	2	SY	11	rs2_16190063	16.01–16.27	6.1E-06	RM5521 (Adachi et al., 2019)
			BJ	236	rs2_16135737	16.01–16.27	7.8E-07	
	*qD_*ada*_2.3*	2	SY	4	rs2_20557721	20.35–20.61	2.3E-06	
	*qD_*ada*_4*	4	SY	2	rs4_19523832	19.28–19.7	2.1E-05	*OsHAK1* (Chen et al., 2017)
			BJ	2	rs4_19545184	19.28–19.7	3.2E-06	
	*qD_*ada*_6*	6	SY	2	rs6_26449847	26.18–26.59	8.3E-06	R32 (Laza et al., 2010)
			BJ	2	rs6_26550078	26.18–26.59	1.9E-05	
	*qD_*ada*_9*	9	BJ	5	rs9_9373482	9.34–9.46	3E-07	
	*qD_*ada*_12*	12	BJ	5	rs12_23157191	23.06–23.28	5.7E-06	
*L*_ada_	*qL_*ada*_9*	9	SY	9	rs9_10813173	10.74–10.85	1.1E-06	
*W*_ada_	*qW_*ada*_1*	1	SY	7	rs1_21260637	21.06–21.35	3.6E-08	
	*qW_*ada*_4*	4	BJ	8	rs4_28730724	28.55–28.89	5E-07	*OsSLAC1* (Kusumi et al., 2012)
	*qW_*ada*_7*	7	SY	2	rs7_24587104	24.51–24.65	1.7E-05	*qHP7b* (Adachi et al., 2019)
			BJ	10	rs7_24591513	24.51–24.65	4.3E-06	*GL7* (Wang et al., 2015)
	*qW_*ada*_11*	11	SY	44	rs11_16428224	16.34–16.51	3E-07	
	*qW_*ada*_12.1*	12	BJ	1	rs12_7814192	7.73–7.93	9.5E-06	
	*qW_*ada*_12.2*	12	BJ	5	rs12_23629169	23.56–23.74	1.3E-06	
*S*_ada_	*qS_*ada*_1*	1	SY	16	rs1_23943199	23.34–23.51	1.2E-06	
	*qS_*ada*_2.1*	2	SY	7	rs2_19733564	19.71–19.89	1.3E-06	
	*qS_*ada*_2.2*	2	SY	38	rs2_20488567	20.35–20.61	2E-07	
			BJ	1	rs2_20365095	20.35–20.61	3.1E-05	
	*qS_*ada*_4.1*	4	SY	2	rs4_14539393	14.35–14.61	6.6E-06	
	*qS_*ada*_4.2*	4	SY	8	rs4_20417902	20.28–20.49	7.2E-07	*OsARVL4* (Wang et al., 2016)
			BJ	1	rs4_20346989	20.28–20.49	2E-05	
	*qS_*ada*_4.3*	4	BJ	1	rs4_34808511	34.79–34.97	7.7E-06	*AM1* (Sheng et al., 2014)
	*qS_*ada*_5*	5	BJ	3	rs5_24737640	24.66–24.99	8.4E-06	
	*qS_*ada*_6*	6	SY	8	rs6_4442388	4.32–4.6	3.7E-06	*OsERF71* (Lee et al., 2016)
	*qS_*ada*_7*	7	SY	28	rs7_26393738	26.32–26.49	6.8E-08	
	*qS_*ada*_9*	9	SY	19	rs9_10813173	10.74–10.85	4.7E-07	
	*qS_*ada*_12*	12	BJ	62	rs12_23600429	23.56–23.74	1.6E-08	
*D*_aba_	*qD_*aba*_1*	1	BJ	1	rs1_23354786	23.34–23.51	4.9E-06	
	*qD_*aba*_2.1*	2	SY	3	rs2_8969123	8.87–9.05	9.8E-06	*OsSPCH2* (Liu et al., 2009);
			BJ	2	rs2_8982952	8.87–9.05	2.3E-05	QTL for △^13^C (Takai et al., 2009)
	*qD_*aba*_2.2*	2	SY	52	rs2_20557721	20.35–20.61	3.2E-07	
	*qD_*aba*_7*	7	SY	11	rs7_15707285	15.68–15.87	1.5E-06	
	*qD_*aba*_9*	9	BJ	2	rs9_9373482	9.34–9.46	2E-06	
*L*_aba_	*qL_*aba*_2*	2	SY	42	rs2_20525345	20.35–20.61	8.6E-08	
	*qL_*aba*_3.1*	3	SY	1	rs3_10158428	10.13–10.39	9E-07	*qHP3a* (Adachi et al., 2019)
	*qL_*aba*_3.2*	3	SY	8	rs3_29815053	29.65–30.06	2E-06	*OsCML4* (Yin et al., 2015)
	*qL_*aba*_4*	4	BJ	1	rs4_20635702	20.6–20.71	1.3E-06	
	*qL_*aba*_6*	6	BJ	3	rs6_27402260	27.37–27.55	1.1E-06	C962 (Laza et al., 2010)
	*qL_*aba*_7*	7	BJ	10	rs7_15859780	15.68–15.87	7.1E-06	
	*qL_*aba*_9*	9	SY	11	rs9_10813173	10.74–10.85	2.9E-06	
*W*_aba_	*qW_*aba*_1*	1	SY	2	rs1_21260637	21.06–21.35	1.4E-06	
	*qW_*aba*_2*	2	SY	24	rs2_20483127	20.35–20.61	1.4E-06	
	*qW_*aba*_3*	3	SY	6	rs3_21383461	21.25–21.45	8.1E-06	QTL for △^13^C (Takai et al., 2009)
	*qW_*aba*_6*	6	BJ	1	rs6_27498819	27.37–27.55	4.3E-06	C962 (Laza et al., 2010)
	*qW_*aba*_7*	7	SY	1	rs7_24591765	24.51–24.65	3.1E-05	*qHP7b* (Adachi et al., 2019)
			BJ	7	rs7_24591513	24.51–24.65	5.6E-06	*GL7* (Wang et al., 2015)
*S*_aba_	*qS_*aba*_1.1*	1	SY	2	rs1_13961260	13.9–14.02	3.7E-07	
	*qS_*aba*_1.2*	1	SY	1	rs1_23500465	23.34–23.51	7.9E-06	
	*qS_*aba*_2.1*	2	SY	29	rs2_19722768	19.71–19.89	2E-06	
	*qS_*aba*_2.2*	2	SY	153	rs2_20448952	20.35–20.61	3.3E-07	
	*qS_*aba*_2.3*	2	SY	40	rs2_30288190	30.05–30.35	5.3E-07	*Ghd2* (Liu et al., 2016)
	*qS_*aba*_3*	3	BJ	2	rs3_5073962	5.06–5.32	1.5E-06	
	*qS_*aba*_4.1*	4	BJ	1	rs4_14525284	14.35–14.61	4.7E-06	
	*qS_*aba*_4.2*	4	SY	7	rs4_20417902	20.28–20.49	5.8E-06	*OsARVL4* (Wang et al., 2016)
			BJ	18	rs4_20304312	20.28–20.49	4.2E-06	
	*qS_*aba*_6.1*	6	SY	4	rs6_4442388	4.32–4.6	9.9E-06	*OsERF71* (Lee et al., 2016)
	*qS_*aba*_6.2*	6	SY	3	rs6_23466436	23.37–23.53	4.3E-06	QTL for △^13^C (Takai et al., 2009)
	*qS_*aba*_6.3*	6	BJ	12	rs6_27378699	27.37–27.55	5.1E-06	C962 (Laza et al., 2010)
	*qS_*aba*_7.1*	7	SY	2	rs7_26032867	26.00–26.25	1.6E-05	*qSf7b* (Zhao et al., 2008);
			BJ	13	rs7_26086686	26.00–26.25	3.3E-06	*OsFLP* (Wu et al., 2019)
	*qS_*aba*_7.2*	7	SY	5	rs7_26449008	26.32–26.49	4.4E-06	
	*qS_*aba*_8.1*	8	SY	3	rs8_4666161	4.58–4.9	5.1E-06	
	*qS_*aba*_8.2*	8	SY	3	rs8_9881223	9.83–10.08	2.6E-06	
	*qS_*aba*_9.1*	9	SY	11	rs9_10812793	10.74–10.85	8.7E-07	
	*qS_*aba*_9.2*	9	SY	6	rs9_14393303	14.17–14.47	1.2E-06	
	*qS_*aba*_10*	10	SY	33	rs10_3553860	3.47–3.6	2.2E-06	
	*qS_*aba*_11*	11	SY	12	rs11_18132552	18.09–18.23	1E-06	*qPn11*, *qGs11*, *qTr11* (Zhao et al., 2008)
	*qS_*aba*_12.1*	12	SY	8	rs12_7220708	7.13–7.25	2.4E-06	
	*qS_*aba*_12.2*	12	BJ	14	rs12_23600429	23.56–23.74	1E-07	

*D_*ada*_, stomatal density on the adaxial surface; D_*aba*_, stomatal density on the abaxial surface; L_*ada*_, guard cell length on the adaxial surface; L_*aba*_, guard cell length on the abaxial surface; W_*ada*_, guard cell width on the adaxial surface; W_*aba*_, guard cell width on the abaxial surface; S_*ada*_, stomatal size on the adaxial surface; S_*aba*_, stomatal size on the abaxial surface; Chr, chromosome; Envir., environment; SY, Sanya; BJ, Beijing; Peak region on the chromosome.*

### Coincidence of QTLs for Stomata-Related Traits on Adaxial and Abaxial Leaf Surfaces

**FIGURE 2:**
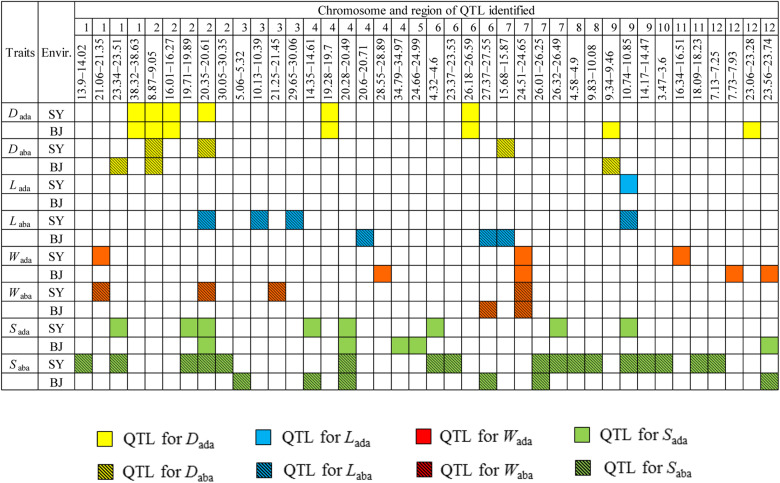
Distribution of quantitative trait loci (QTLs) associated with stomata-related traits on abaxial and adaxial leaf surfaces across the two environments on 12 chromosomes according to physical distance. *D*_ada_, stomatal density on the adaxial surface; *D*_aba_, stomatal density on the abaxial surface; *L*_ada_, guard cell length on the adaxial surface; *L*_aba_, guard cell length on the abaxial surface; *W*_ada_, guard cell width on the adaxial surface; *W*_aba_, guard cell width on the abaxial surface; *S*_ada_, stomatal size on the adaxial surface; *S*_aba_, stomatal size on the abaxial surface; Envir., environment; SY, Sanya; BJ, Beijing.

Comparisons of 26 QTLs affecting stomata-related traits at the adaxial surface with 38 QTLs at the abaxial surface demonstrated that 12 QTL regions were simultaneously detected for the same stomata-related traits at both leaf surfaces in the same environment. These regions included two regions (8.87–9.05 Mb on chromosome 2 harboring *qD_*ada*_2.1* and *qD_*aba*_2.1*, and 9.34–9.46 Mb on chromosome 9 harboring *qD_*ada*_9* and *qD_*aba*_9*) for *D*, six regions (23.34–23.51 Mb on chromosome 1 harboring *qS_*ada*_1* and *qS_*aba*_1.2*, 19.71–19.89 Mb on chromosome 2 harboring *qS_*ada*_2.1* and *qS_*aba*_2.1*, 20.28–20.49 Mb on chromosome 4 harboring *qS_*ada*_4.2* and *qS_*aba*_4.2*, 4.32–4.60 Mb on chromosome 6 harboring *qS_*ada*_6* and *qS_*aba*_6.1*, 26.32–26.49 Mb on chromosome 7 harboring *qS_*ada*_7* and *qS_*aba*_7.2*, and 23.56–23.74 Mb on chromosome 12 harboring *qS_*ada*_12* and *qS_*aba*_12.2*) for *S*, two regions (21.06–21.35 Mb on chromosome 1 harboring *qW_*ada*_1* and *qW_*aba*_1*, and 24.51–24.65 Mb on chromosome 7 harboring *qW_*ada*_7* and *qW_*aba*_7*) for *W*, one region (10.74–10.85 Mb on chromosome 9 harboring *qL_*ada*_9*, *qL_*a*__*b*__*a*_9*, *qS_*aba*_9.1*, and *qS_*ada*_9*) affecting *L* and *S*, and one region (20.35–20.61 Mb on chromosome 2 harboring *qD_*ada*_2.3*, *qD_*a*__*b*__*a*_2.2*, *qS_*ada*_2.2*, and *qS_*a*__*b*__*a*_2.2*) for *D* and *S* (Figure 2), which probably explain the genetic basis of the significant correlations of stomata-related traits at the two leaf surfaces.

### Candidate Gene Identification of the Important QTLs

For the eight stomata-related traits, we conducted haplotype analyses to identify candidate genes for the 12 QTLs consistently identified in the nine chromosomal regions across the two environments (Table 1). In addition, considering that the eight stomata-related traits were significantly different between *xian* and *geng* in the two environments, except *D*_ada_ and *L*_ada_ in BJ (Figure 1), we conducted haplotype analysis of the targeted genes associated with the eight stomata-related traits in whole population, and the *xian* and *geng* subpopulations. A total of 315 annotated genes located in the nine chromosomal regions were selected for haplotype analysis according to the Rice Annotation Project Database, and 64 genes were finally considered as candidate genes (Supplementary Table S4).

**FIGURE 3:**
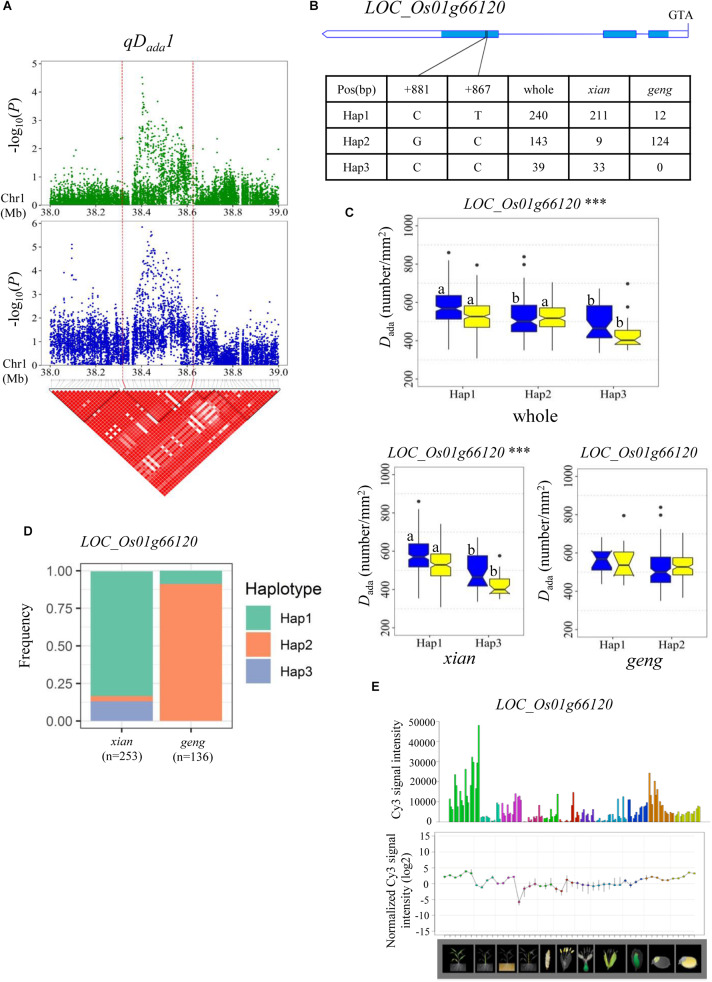
Haplotype analyses of the *LOC_Os01g66120* candidate gene for the *D*_ada_ at *qD_*ada*_1*. **(A)** Linkage disequilibrium (LD) block surrounding the peak on chromosome 1. Dashed lines indicate the candidate region for the peak. **(B)** Gene structure of *LOC_Os01g66120* and DNA polymorphisms in that gene. **(C)** Boxplots for stomatal density on the adaxial surface (*D*_ada_) based on haplotypes for *LOC_Os01g66120* using non-synonymous single nucleotide polymorphisms (SNPs) within the coding region in the whole, *xian*, and *geng* populations. ***Denotes the significance of ANOVA at *P* < 0.001. Letters on histograms (a and b) are ranked by Duncan’s test at *P* < 0.05. Blue and yellow colors indicate Sanya and Beijing, respectively. **(D)** Frequencies of different haplotypes of the *LOC_Os01g66120* in *xian* and *geng* subpopulations. **(E)** Spatio-temporal expression of *LOC_Os01g66120* in various Nipponbare tissues throughout the entire growth period in the field [downloaded from RiceXPro (version 3.0)].

**FIGURE 4:**
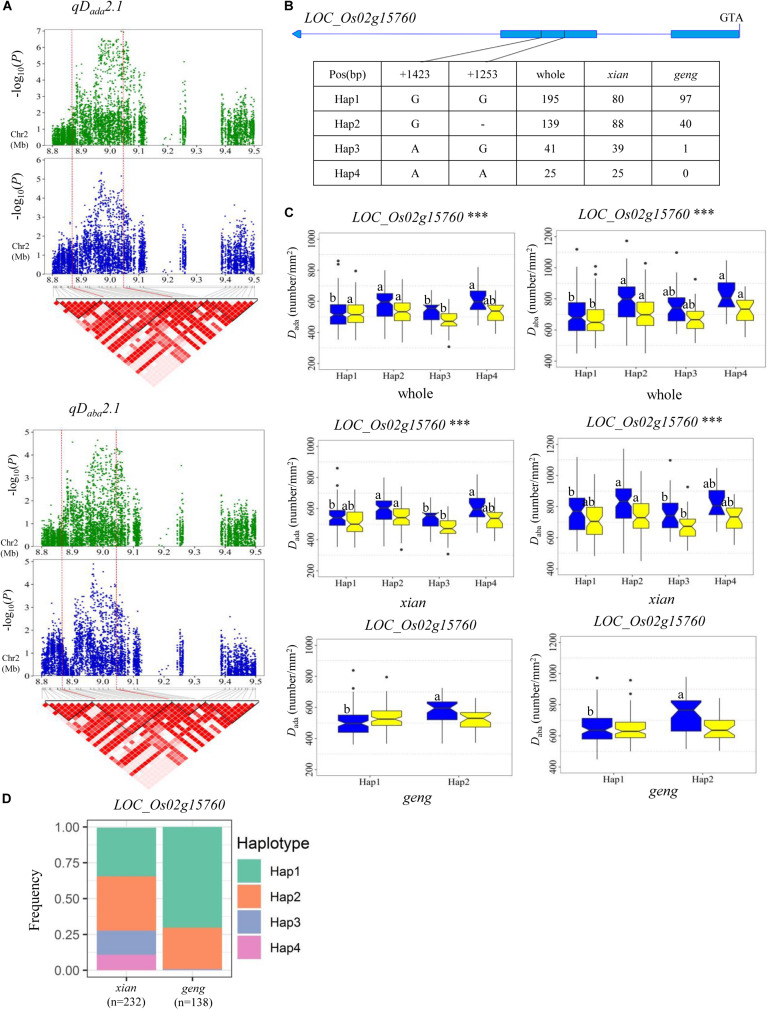
Haplotype analyses for the *D*_ada_ and *D*_aba_ of the *LOC_Os02g15760* candidate gene at *qD_*ada*_2.1* and *qD_*aba*_2.1*. **(A)** Linkage disequilibrium (LD) block surrounding the peak on chromosome 2. Dashed lines indicate the candidate region for the peak. **(B)** Gene structure of *LOC_Os02g15760* and DNA polymorphisms in that gene. **(C)** Boxplots for stomatal density on the adaxial surface (*D*_ada_) and the abaxial surface (*D*_aba_) based on haplotypes for the *LOC_Os02g15760* using non-synonymous SNPs within the coding region in the whole, *xian*, and *geng* populations. ***Denotes the significance of ANOVA at *P* < 0.001. Letters on histograms (a and b) are ranked by Duncan’s test at *P* < 0.05. Blue and yellow colors indicate Sanya and Beijing, respectively. **(D)** Frequencies of different haplotypes of *LOC_Os02g15760* in *xian* and *geng* subpopulations.

For *D*_ada_ and *D*_*a*__*b*__*a*_, haplotype analysis revealed 7, 9, 11, 5, and 5 candidate genes for *qD_*ada*_1*, *qD_*ada*_2.1*/*qD_*aba*_2.1*, *qD_*ada*_2.2*, *qD_*ada*_4*, and *qD_*ada*_6*, respectively (Supplementary Table S4). In the region from 38.32 to 38.63 Mb on chromosome 1 harboring *qD_*ada*_1* (Figure 3A), 45 annotated genes were selected based on the Nipponbare reference genome IRGSP 1.0 for haplotype analysis with all non-synonymous SNPs inside of the gene coding regions in the whole, *xian*, and *geng* populations. Highly significant differences in *D*_ada_ were detected among different haplotypes of the seven candidate genes (*LOC_Os01g66120*, *LOC_Os01g66130*, *LOC_Os01g66180*, *LOC_Os01g66230*, *LOC_Os01g66240*, *LOC_Os01g66250*, and *LOC_Os01g66260*) (Supplementary Table S4 and Figure 3). Among them, *LOC_Os01g66120* is identical to *OsNAC6*, which is a member of the NAC transcription factor gene family in rice (Table 2; Nakashima et al., 2007). Three haplotypes were found at *OsNAC6* and the frequencies of them were significantly associated with the rice subpopulations according to Fisher’s exact tests (Supplementary Table S5). Haplotype (Hap) 1 (CT) exhibited significantly higher *D*_ada_ than Hap3 (CC) in the whole and *xian* populations in the two environments (Figures 3B,C). In addition, 87.9% of the accessions with the high-*D*_*ada*_ Hap1 (*n* = 211) and 84.6% of the accessions with the low-*D*_*ada*_ Hap3 (*n* = 33) belonged to the *xian* subpopulation (Figure 3D). *OsNAC6* is relatively highly expressed in specific organs (leaf blade, ovary, embryo, and endosperm) according to a publicly available rice gene expression profile database [RiceXPro (version 3.0)] (Figure 3E). In the region from 8.87 to 9.05 Mb on chromosome 2 harboring *qD_*ada*_2.1* and *qD_*aba*_2.1* (Figure 4A), highly significant differences in the mean *D*_ada_ and *D*_aba_ were detected among different haplotypes of the nine candidate genes (Supplementary Table S4 and Figure 4). Among them, *LOC_Os02g15760* is identical to a *SPCH* transcriptional factor gene *OsSPCH2*, which regulates the initiation of stomatal lineage, as previously reported (Liu et al., 2009; Wu et al., 2019; Table 2). Haplotype analysis revealed that Hap2 was significantly associated with larger *D*_aba_ and *D*_aba_ than Hap3 in the *xian* population (Figures 4B,C). In contrast, 63.3% of the accessions with the high-*D*_*ada*_ Hap2 and 95.1% of the accessions with the low-*D*_*ada*_ Hap3 belonged to the *xian* subpopulation (Figure 4D). The frequency distributions of Hap3 significantly differed between the *xian* and *geng* subgroups, whereas the frequency distributions of Hap2 did not significantly differ between the *xian* and *geng* subgroups (Supplementary Table S5). In addition, 11, 5, and 5 candidate genes for *qD_*ada*_2.2*, *qD_*ada*_4*, and *qD_*ada*_6*, respectively, were identified with significant differences in the mean *D*_ada_ among the different haplotypes based on haplotype analysis (Supplementary Table S4).

**TABLE 2:** List of six the most likely candidate genes for five important quantitative trait loci (QTLs) associated with stomata-related traits.

**QTL**	**Candidate gene**	**MSU_Locus_Annotation**	**Known function**
*qD_*ada*_1*	*LOC_Os01g66120*	No apical meristem protein, putative, expressed	A member of the NAC transcription factor gene family in rice
*qD_*ada*_2.1*/ *qD_*aba*_2.1*	*LOC_Os02g15760* (*OsSPCH2*)	Helix-loop-helix DNA-binding domain containing protein, expressed	Regulates the initiation of stomatal lineage in rice
*qS_*ada*_2.2*	*LOC_Os02g34320*	Basic helix-loop-helix, putative, expressed	–
*qS_*aba*_7.1*	*LOC_Os07g43420* (*OsFLP*)	R2R3 MYB transcription factor, putative, expressed	Negatively regulates guard mother symmetric cell division in rice
	*LOC_Os07g43530*	Helix-loop-helix DNA-binding domain containing protein, expressed	–
*qW_*ada*_7*/*qW_*aba*_7*	*LOC_Os07g41200* (*GL7*)	Expressed protein	Regulates longitudinal cell elongation, contributing to an increase in grain length and improvement of grain appearance quality

**FIGURE 5:**
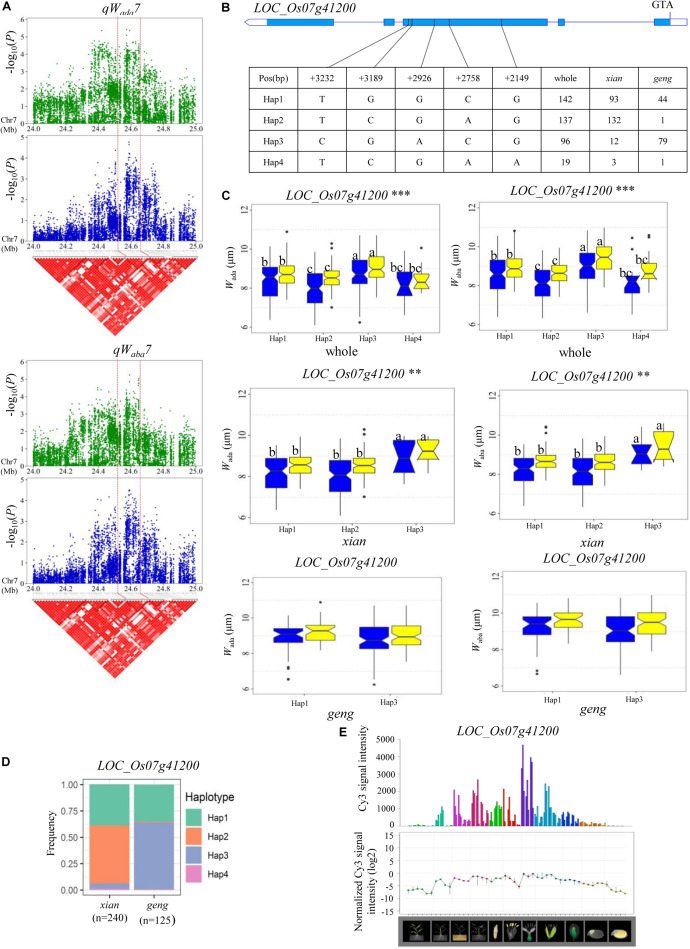
Haplotype analyses for the *W*_ada_ and *W*_aba_ of the *LOC_Os07g41200* candidate gene at *qW_*ada*_7* and *qW_*aba*_7*. **(A)** Linkage disequilibrium (LD) block surrounding the peak on chromosome 7. Dashed lines indicate the candidate region for the peak. **(B)** Gene structure of *LOC_Os07g41200* and DNA polymorphisms in that gene. **(C)** Boxplots for guard cell width on the adaxial surface (*W*_ada_) and the abaxial surface (*W*_aba_) based on haplotypes for *LOC_Os07g41200* using non-synonymous SNPs within the coding region in the whole, *xian*, and *geng* populations. ** and ***denotes the significance of ANOVA at *P* < 0.01 and *P* < 0.001, respectively. Letters on histograms (a, b, and c) are ranked by Duncan’s test at *P* < 0.05. Blue and yellow colors indicate Sanya and Beijing, respectively. **(D)** Frequencies of different haplotypes of *LOC_Os07g41200* in *xian* and *geng* subpopulations. **(E)** Spatio-temporal expression of *LOC_Os07g41200* in various Nipponbare tissues throughout the entire growth period in the field [downloaded from RiceXPro (version 3.0)].

For *W*_ada_ and *W*_*a*__*b*__*a*_, a high peak of *qW_*ada*_7* on chromosome 7 was mapped together with the peak of *qW_*a*__*b*__*a*_7*. A total of 19 annotated genes were selected from the region of 24.51–24.65 Mb (140 kb) (Figure 5A), and 11 candidate genes were identified with significant differences in the mean *W*_ada_ and *W*_*a*__*b*__*a*_ among the different haplotypes (Supplementary Table S4 and Figure 5). Among them, *LOC_Os07g41200* is identical to *Grain Length on Chromosome 7* (*GL7*), which regulates longitudinal cell elongation, contributing to an increase in grain length and improvement of grain appearance quality (Wang et al., 2015; Table 2). Hap3 showed significantly larger *W*_ada_ and *W*_*a*__*b*__*a*_ values than Hap1 and Hap2 in the whole and *xian* populations (Figures 5B,C). We determined that 82.3% of the accessions with high-*W*_aba_ Hap3 belonged to the *geng* subpopulation, whereas 65.5 and 96.4% with low-*W*_aba_ Hap1 and Hap2, respectively, belonged to the *xian* subpopulation (Figure 5D). The frequency distributions of Hap2 and Hap3 significantly differed between the *xian* and *geng* subgroups, whereas the frequency distribution of Hap1 did not significantly differ between the two subgroups (Supplementary Table S5). *GL7* is relatively highly expressed in some specific organs except leaf blade, embryo, and endosperm according to rice gene expression profile database [RiceXPro (version 3.0)] (Figure 5E).

**FIGURE 6:**
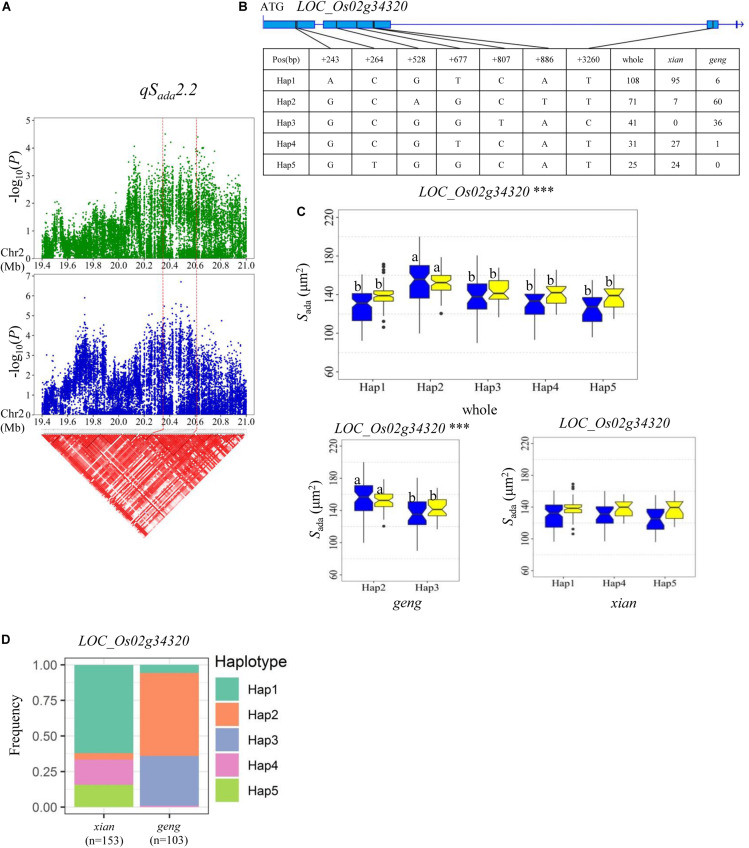
Haplotype analyses for *S*_a__d__a_ of *LOC_Os02g34320* candidate gene at *qS_*a*__*d*__*a*_2.2*. **(A)** Linkage disequilibrium (LD) block surrounding the peak on chromosome 2. Dashed lines indicate the candidate region for the peak. **(B)** Gene structure of *LOC_Os02g34320* and DNA polymorphisms in that gene. **(C)** Boxplots for stomatal size on the adaxial surface (*S*_ada_) based on haplotypes for *LOC_Os02g34320* using non-synonymous SNPs within the coding region in the whole, *xian* and *geng* populations. ***Denotes significance of ANOVA at *P* < 0.001. Letters on histograms (a and b) are ranked by Duncan’s test at *P* < 0.05. Blue and yellow colors indicate Sanya and Beijing, respectively. **(D)** Frequencies of different haplotypes of *LOC_Os02g34320* in *xian* and *geng* subpopulations.

Regarding *S*_ada_ and *S*_aba_, haplotype analysis revealed that four, eight, and four candidate genes were identified for *qS_*ada*_2.2*, *qS_*ada*_4.2*/*qS_*aba*_4.2*, and *qS_*aba*_7.1*, respectively (Supplementary Table S4). *QS_*ada*_2.2* was identified in the region of 20.35–20.61 Mb on chromosome 2 (Figure 6A), which contains 33 annotated genes. Four candidate genes (*LOC_Os02g34260*, *LOC_Os02g34270*, *LOC_Os02g34320*, and *LOC_Os02g34410*) were detected with significant differences in the mean *S*_ada_ among the different haplotypes (Supplementary Table S4 and Figure 6). Among them, *LOC_Os02g34320* encodes a basic helix-loop-helix protein (Table 2). Five haplotypes were detected at *LOC_Os02g34320* and the frequency distributions of them significantly differed between the *xian* and *geng* subgroups (Supplementary Table S5). Hap2 exhibited a significantly higher *S*_ada_ value than the other four haplotypes in the whole population (Figures 6B,C). 84.5% of the accessions with the high-*S*_ada_ Hap2 belonged to the *geng* subpopulation, 88, 87.1, and 96% of the accessions with the low-*S*_ada_ Hap1, Hap4 and Hap5, respectively, all belonged to the *xian* subgroup, and 87.8% of the accessions with the low-*S*_ada_ Hap3 belonged to the *geng* subpopulation (Figure 6D). Moreover, Hap2 showed a significantly higher *S*_ada_ value than Hap3 in the *xian* population in both environments (Figure 6C).

**FIGURE 7:**
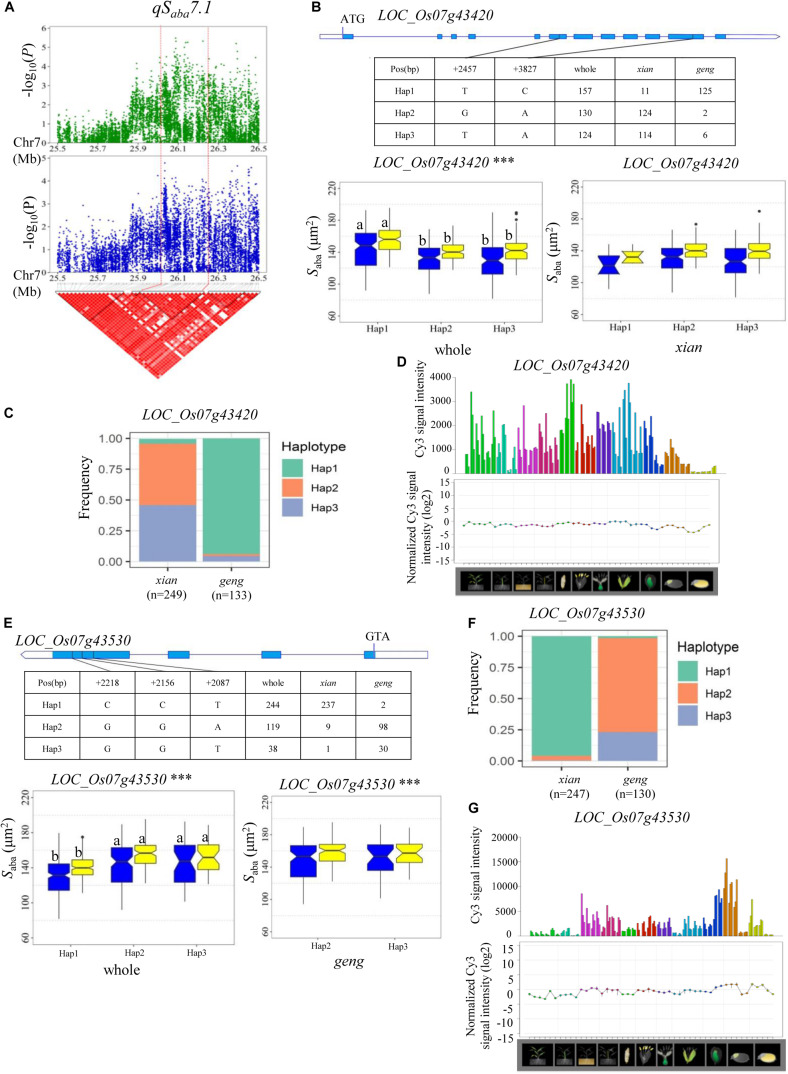
Haplotype analyses for the *S*_aba_ of the *LOC_Os07g43420* and *LOC_Os07g43530* candidate genes at *qS_*aba*_7.1*. **(A)** Linkage disequilibrium (LD) block surrounding the peak on chromosome 7. Dashed lines indicate the candidate region for the peak. **(B)** Gene structure of *LOC_Os07g43420*, DNA polymorphisms in the gene, and boxplots for stomatal size on the abaxial leaf surface (*S*_aba_) based on haplotypes for *LOC_Os07g43420* using non-synonymous SNPs within the coding region in the whole and *xian* populations. **(C)** Frequencies of different haplotypes of *LOC_Os07g43420* in *xian* and *geng* subpopulations. **(D)** Spatio-temporal expression of *LOC_Os07g43420* in various Nipponbare tissues throughout the entire growth period in the field [downloaded from RiceXPro (version 3.0)]. **(E)** Gene structure of *LOC_Os07g43530*, DNA polymorphisms in the gene, and boxplots for *S*_aba_ based on haplotypes for *LOC_Os07g43530* using non-synonymous SNPs within the coding region in the whole and *geng* populations. ***Denotes significance of ANOVA at *P* < 0.001. Letters on histograms (a and b) are ranked by Duncan’s test at *P* < 0.05. Blue and yellow colors indicate Sanya and Beijing, respectively. **(F)** Frequencies of different haplotypes of *LOC_Os07g43530* in *xian* and *geng* subpopulations. **(G)** Spatio-temporal expression of *LOC_Os07g43530* in various Nipponbare tissues throughout the entire growth period in the field [downloaded from RiceXPro (version 3.0)].

A QTL cluster (*qS_*ada*_4.2* and *qS_*aba*_4.2*) affecting *S*_a__d__a_ and *S*_aba_ was identified in the region of 20.28–20.49 Mb on chromosome 4 in the two environments, containing 28 annotated genes. Among them, eight candidate genes (*LOC_Os04g32160*, *LOC_Os04g32170*, *LOC_Os04g32200*, *LOC_Os04g32210*, *LOC_Os04g32320*, *LOC_Os04g32380*, *LOC_Os04g32480*, and *LOC_Os04g32570*) were identified with significant differences in *S*_a__d__a_ and *S*_aba_ values among the different haplotypes (Supplementary Table S4). In addition, *qS_*aba*_7.1* was fine-mapped to the region of 26.00–26.25 Mb on chromosome 7 (Figure 7A) which harbored 39 annotated genes. Highly significant differences in *S*_aba_ were detected among different haplotypes for four candidate genes (*LOC_Os07g43420*, *LOC_Os07g43510*, *LOC_Os07g43530*, and *LOC_Os07g43580*) (Supplementary Table S4 and Figure 7). Among them, *LOC_Os07g43530* encodes a basic helix-loop-helix protein, while *LOC_Os07g43420* is identical to *OsFLP*, which negatively regulates guard mother symmetric cell division in rice (Wu et al., 2019) (Table 2). Haplotype analysis of *LOC_Os07g43420* revealed that Hap1 was significantly associated with a larger *S*_aba_ value than Hap2 and Hap3 in the whole population (Figure 7B). The frequency distributions of these three haplotypes significantly differed between the *xian* and *geng* subgroups (Supplementary Table S5). Moreover, 79.6% of the accessions with the high-*S*_*aba*_ Hap1 belonged to the *geng* subpopulation, whereas 95.4 and 91.9% of the accessions with the low-*S*_*aba*_ Hap2 and Hap3, respectively, belonged to the *xian* subpopulation (Figure 7C). *OsFLP* is relatively highly expressed in some specific organs except endosperm based on rice gene expression profile database [RiceXPro (version 3.0)] (Figure 7D). For *LOC_Os07g43530*, Hap2 and Hap3 of *LOC_Os07g43530* exhibited a significantly larger *S*_aba_ value than Hap1 in the whole population (Figure 7E). The frequency distributions of these three haplotypes differed significantly between the rice subgroups (Supplementary Table S5). In contrast, 82.3 and 78.9% of the accessions with the low-*S*_*aba*_ Hap2 and Hap3, respectively, belonged to the *xian* subpopulation, whereas 97.1% of the accessions with the high-*S*_*aba*_ Hap1 belonged to the *geng* subpopulation (Figure 7F). *LOC_Os07g43530* is relatively highly expressed in some specific organs except leaf blade according to rice gene expression profile database [RiceXPro (version 3.0)] (Figure 7G).

## Discussion

### Characteristics of Stomata Between *Xian* and *Geng* and Their Responses to the Environment

Asian cultivated rice (*Oryza sativa* L.) is classified into two subspecies, namely *xian* and *geng*. There are significant differences in many morphological and physiological traits associated with evolution of *xian*-*geng*, resulting in many distinguishing features for the two subspecies of cultivated rice (Morishima and Oka, 1981). Generally, *xian* varieties tend to have higher *D* and smaller *S* values than *geng* varieties (Laza et al., 2010). In our present study, *xian* cultivars exhibited significantly higher *D* and smaller *S* values than *geng* cultivars on both the adaxial and abaxial leaf surfaces in two environments, except for *D*_ada_ in BJ (Figure 1A). In addition, the frequencies of most haplotypes at six representative candidate genes for the stomatal-related traits were significantly associated with the rice subpopulations according to Fisher’s exact tests (Supplementary Table S5), suggesting *D* and *S* are related to differentiation of *xian* and *geng* and dependent of population structure. Compared with *geng* varieties, the higher *D* of *xian* varieties was significantly associated with high photosynthetic abilities (Weng and Chen, 1988), most probably because smaller and more numerous stomata can potentially improve stomatal conductance (Zhang et al., 2019). The leaf transpiration efficiency is regulated mainly by stomatal movement, but is also affected by stomatal size and density (Nir et al., 2014). Peng et al. (1998) demonstrated that *xian* cultivars exhibited 25–30% lower transpiration efficiency (ratio of photosynthesis to transpiration) compared with that of the *geng* cultivars. Therefore, the higher photosynthetic rate of *xian* cultivars might be mainly attributed to its higher *D* and lower gas diffusion resistance.

Stomatal density is suggested to be an adaptive mechanism in plants to environmental stresses such as light intensity, water status, and CO_2_ levels (Kondamudi et al., 2018). In this study, the abaxial surface possessed more stomata than the adaxial surface (Supplementary Figure S2), which was in agreement with previous studies (Ishimaru et al., 2001; Laza et al., 2010). Increases in light intensities produced greater increases in *D* for the abaxial surface than for the adaxial surface in *Arabidopsis* (Schlüter et al., 2003). In the present study, 451 rice accessions were grown in May in BJ and December in SY. During the later growth period, the light intensity in SY was stronger than that in BJ. With the increase in light supply during growth from BJ to SY, approximately 64% of the accessions (including about 40% *geng* and 60% *xian* accessions) exhibited parallel increases in *D* and decreases in *S* on both the adaxial and abaxial leaf surfaces, where the abaxial surface increased by an average of 8.3%, which was markedly higher than the average 5.9% of the adaxial surface (Supplementary Table S1). Specifically, *xian* accessions, on average, exceeded 10.3 and 9% of *D*_aba_ and *D*_ada_ in SY compared with that in BJ, whereas *geng* accessions showed only 4.7 and 0.5% of *D*_aba_ and *D*_ada_ higher values in SY than in BJ, respectively (Supplementary Table S1). The phenomenon might mainly be attributed to different adaptations to the environments, especially the light intensity on stomata between *xian* and *geng*. *Xian* varieties are generally adapted to tropical lowland cultivation with higher light intensity, whereas most *geng* varieties are adapted to temperate climates (Khush, 1997).

### Comparison of QTLs Detected in This Study With Previously Reported QTLs and Cloned Genes

Of the 64 QTLs for stomata-related traits identified in this study, seven were located in the same or adjacent regions containing previously reported QTLs and cloned genes affecting stomata-related traits in rice (Table 1). For example, *qD_*ada*_2.1* for *D*_ada_, and *qD_*aba*_2.1* for *D*_aba_ in the region 8.87–9.05 Mb on chromosome 2, which was co-located with *OsSPCH2* regulating the initiation of stomatal lineage in rice (Liu et al., 2009); *qD*_*ada*_6 affecting *D*_ada_ in the region of 26.18–26.59 Mb on chromosome 6, which harbored a previously reported QTL for *D*_ada_ identified on chromosome 6 near marker R32 (Laza et al., 2010); *qL_*aba*_6* for *L*_*a*__*b*__*a*_, *qW_*aba*_6* for *W*_aba_, and *qS_*aba*_6.3* for *S*_*a*__*b*__*a*_, located in the region of 27.37–27.55 Mb on chromosome 6, are co-located with a QTL for *S*_*a*__*b*__*a*_ on chromosome 6 that overlapped marker C962 (Laza et al., 2010); *qS_*aba*_7.1* affecting *S*_aba_ was mapped in the region of 26.00–26.25 Mb on chromosome 7, which harbored the previously reported *qSf7b* for leaf stomata frequency (Zhao et al., 2008) and *OsFLP*, which negatively regulates guard mother symmetric cell division in rice (Wu et al., 2019).

Many stomata-related traits are associated with photosynthesis and drought stress responsive traits (Franks and Beerling, 2009; Xu et al., 2016; Zandalinas et al., 2018). In this study, 12 and 7 QTLs for stomata-related traits were identified in the same or adjacent regions with previously reported QTLs or cloned genes affecting photosynthesis and drought stress responsive traits, respectively (Table 1). These 12 QTLs included *qD_*ada*_2.1* for *D*_ada_ and *qD_*aba*_2.1* for *D*_aba_ in the region of 8.87–9.05 Mb on chromosome 2, *qW_*aba*_3* for *W*_aba_ in the region of 21.25–21.45 Mb on chromosome 3, and *qS_*aba*_6.2* for *S*_aba_ in the region of 23.37–23.53 Mb on chromosome 6, which mapped together with three QTLs controlling carbon isotope discrimination, which contributed to stomatal conductance (Takai et al., 2009); *qS_*ada*_4.2* for *S*_ada_ and *qS_*aba*_4.2* for *S*_aba_ in the region of 20.28–20.49 Mb on chromosome 4 mapped together with *abaxial rolling and vein-albino leaves 4* (*OsARVL4*), which influences leaf morphology and the photosynthetic rate (Wang et al., 2016); *qW_*ada*_7* for *W*_ada_ and *qW_*a*__*b*__*a*_7* for *W*_*a*__*b*__*a*_ in the region of 24.51–24.65 Mb on chromosome 7, *qL_*aba*_3.1* for *L*_aba_ in the region of 10.13–10.39 Mb on chromosome 3, and *qD_*ada*_2.2* for *D*_ada_ in the region of 16.01–16.27 Mb on chromosome 2 were mapped together with *qHP7b*, *qHP3a*, and a QTL near marker RM5521 controlling leaf photosynthesis, respectively (Adachi et al., 2019); *qW_*ada*_4* for *W*_ada_ in the region of 28.55–28.89 Mb on chromosome 4 mapped together with *SLOW ANION CHANNEL-ASSOCIATED 1* (*OsSLAC1*), which is responsible for increased leaf photosynthesis caused by elevated stomatal conductance in rice (Kusumi et al., 2012); *qS_*aba*_11* for *S*_aba_ in the region 18.09–18.23 Mb on chromosome 11 mapped together with a QTL cluster harboring *qPn11*, *qGs11*, and *qTr11*, which affect the net photosynthetic rate, stomata conductance, and the transpiration rate, respectively (Zhao et al., 2008).

Another seven QTLs included *qD_*ada*_1* for *D*_ada_ in the region of 38.32–38.63 Mb on chromosome 1 and *qD_*ada*_4* for *D*_ada_ in the region of 19.28–19.7 Mb on chromosome 4 mapped together with *OsNAC6* (Nakashima et al., 2007) and a High-affinity potassium transporter *OsHAK1* (Chen et al., 2017), which are associated with rice drought tolerance; *qS_*ada*_4.3* for *S*_ada_ in the region of 34.79–34.97 Mb on chromosome 4 mapped in the adjacent region to *Albino midrib 1* (*AM1*), which is associated with chloroplast development and drought tolerance in rice (Sheng et al., 2014); *qS_*ada*_6* for *S*_ada_, and *qS_*aba*_6.1* for *S*_aba_ in the region of 4.32–4.6 Mb on chromosome 6 mapped together with a drought-responsive AP2/ERF transcription factor *OsERF71*, which is associated with root structure and drought resistance of rice (Lee et al., 2016); *qS_*aba*_2.3* for *S*_aba_ in the region of 30.05–30.35 Mb on chromosome 2 mapped together with *Grain number, plant height, and heading date2* (*Ghd2*), a CONSTANS-like gene that influences drought sensitivity via regulation of senescence in rice (Liu et al., 2016); and *qL_*aba*_3.2* for *L*_aba_ in the region of 29.65–30.06 Mb on chromosome 3 mapped together with a Calmodulin-like gene *OsCML4* for drought tolerance through scavenging of reactive oxygen species in rice (Yin et al., 2015). Allelism of the above QTLs for stomata-related traits identified in this study with previously reported QTLs or genes requires further verification via fine mapping and QTL cloning.

### Most Likely Candidate Genes for the Important QTLs

GWAS and haplotype analysis of candidate genes revealed 64 candidate genes governing the 12 stable QTLs in nine chromosomal regions that were consistently detected in both environments. Based on the functional annotation of candidate genes, we speculated on the most likely candidate genes of *qD_*ada*_1*, *qD_*ada*_2.1*/ *qD_*a*__*b*__*a*_2.1*, *qS_*aba*_7.1*, *qS_*ada*_2.2*, and *qW*_a__d__a_*7*/*qW*_aba_*7*.

The region 38.32–38.63 Mb on chromosome 1, harboring *qD_*ada*_1*, contains seven candidate genes, including *OsNAC6* (*LOC_Os01g66120*), a member of the NAC transcription factor gene family in rice (Nakashima et al., 2007). Transgenic rice plants overexpressing *OsNAC6* showed an improved tolerance to dehydration and high-salt stresses, and exhibited increased tolerance to blast disease (Nakashima et al., 2007). Many previous studies indicated that plant *D* plays an important role in plant responses to salt (Wei et al., 2019) and drought stress (Zandalinas et al., 2018). Moreover, water deficit leads to an increase in *D* and a decrease in *S* (Xu and Zhou, 2008), indicating that this may be partially attributed to improving the adaptation of plants to drought. In the present study, Hap1 (CT) exhibited a significantly higher *D*_ada_ value than Hap3 (CC) in the whole and *xian* populations in the two environments (Figure 3C). *OsNAC6* is highly expressed in the leaf blade according to a publicly available rice gene expression profile database [RiceXPro (version 3.0)] (Figure 3D). For this reason, *OsNAC6* (*LOC_Os01g66120*) is considered the most likely candidate gene of *qD_*ada*_1*.

A QTL cluster (*qD_*ada*_2.1* and *qD_*a*__*b*__*a*_2.1*) was identified in the region of 8.87–9.05 Mb on chromosome 2, with the most significant associated SNP (rs2_8970096, *P* = 1.0 × 10^–7^) being detected close (approximately 87 kb downstream) to stomata-related gene *OsSPCH2*. A previous study demonstrated that *OsSPCH2* regulates the initiation of stomatal lineage in rice (Liu et al., 2009; Wu et al., 2019). Compared with the wild-type plant, the stomatal pattern and morphogenesis of knock-out mutants of *OsSPCH2* (*c-osspch2*) were identical to those of the wild-type plants; however, the *D* value in *c-osspch2* was significantly reduced (Wu et al., 2019). In this study, four haplotypes of *OsSPCH2* were found, with Hap 2(G-) showing significantly higher *D*_ada_ and *D*_aba_ values than those of Hap 3(AG) in the *xian* subpopulation (Figure 4C). We considered that *OsSPCH2* (*LOC_Os02g15760*) is the most likely candidate gene of *qD_*ada*_2.1* and *qD_*aba*_2.1*.

On chromosome 7, a high peak of *qW*_a__d__a_*7* was mapped together with the peak of *qW*_aba_*7*, containing 11 candidate genes. Among them, *Grain Length on Chromosome 7* (*GL7*, *LOC_Os07g41200*), encodes a protein homologous to *Arabidopsis thaliana* LONGIFOLIA proteins, which regulate longitudinal cell elongation, contributing to an increase in grain length and an improvement of grain appearance quality (Wang et al., 2015). Compared with Hap3, Hap1, and Hap 2 both decreased *W* on the adaxial and abaxial leaf surfaces in the whole and *xian* populations in the two environments (Figure 5C). This suggested that *GL7* probably affects guard cell width in rice. Reverse genetic approaches might be used to confirm whether *GL7* is the candidate gene of *qW*_a__d__a_*7* and *qW*_aba_*7*.

For *qS_*ada*_2.2*, *LOC_Os02g34320*, encoding a basic helix_loop_helix transcription factor, is the most likely candidate gene, with significant differences in the *S*_ada_ value among different haplotypes (Figure 6C). In *Arabidopsis*, three basic helix_loop_helix transcription factors, *SPEECHLESS* (*SPCH*), *MUTE* and *FAMA*, are essential for the initiation of stomatal lineage, termination of meristemoid fate, and the transition to guard mother cell identity, respectively (Pillitteri et al., 2007; Wu et al., 2019). Previous work indicated that *OsSPCH2* (*LOC_Os02g15760*), encoding a helix_loop_helix transcription factor, controls the initiation of stomatal files in rice (Wu et al., 2019). In the present study, five haplotypes of *LOC_Os02g34320* were detected in the whole population, with Hap 2 being associated with a significantly larger *S*_ada_ value than the other four haplotypes in both environments (Figure 6C). In addition, Hap 2 had a significantly larger *S*_ada_ value than Hap3 in the *geng* subpopulation, suggesting that *LOC_Os02g34320* is a likely candidate gene of *qS_*ada*_2.2* that probably affects *S*_ada_ in rice (Figure 6).

Of the four candidate genes for *qS_*aba*_7.1*, *LOC_Os07g43420* (*OsFLP*), which encodes a functionally redundant R2R3 MYB transcription factor, negatively regulates guard mother symmetric cell division in rice (Wu et al., 2019) and is the most likely candidate gene. In addition, *LOC_Os07g43530*, a basic helix_loop_helix transcription factor gene, is also a likely candidate gene of *qS_*aba*_7.1*. In this study, no significant difference in the two genes was detected among different haplotypes in the *xian*/*geng* subpopulations, or only one prevalent haplotype (contained in more than 10 accessions) was observed in the *xian*/*geng* subpopulations, although there were significant differences for *S*_aba_ between different haplotypes in the whole population (Figures 7B,E). This indicated that *xian*-*geng* differentiation probably led to significant phenotypic differences in *S*_aba_ among different haplotypes. Thus, further study is required to validate whether *LOC_Os07g43530* and *OsFLP* are candidate genes for *qS_*aba*_7.1* or evolution-related genes of *xian* and *geng*. The above promising candidate genes associated with the eight rice stomata-related traits are valuable resources for future functional characterization and marker-assisted breeding to improve rice grain yield under non-stressed and abiotic stressed conditions after functional tests using transformation or CRISPR/Cas9 genome editing.

### Application in Rice Breeding for High Yield Potential Under Non-stressed and Abiotic Stressed Conditions

Under well-watered conditions, an increase in *D* could allow plants to improve stomatal conductance for gas exchange on the leaf surface, thus avoiding photosynthetic limitation by CO_2_ supply. Positive correlations between stomatal conductance and *D* have been reported in previous studies (Xu and Zhou, 2008; Zhao et al., 2008). Schlüter et al. (2003) revealed that only an increase in stomatal density without altering any other internal leaf architecture could improve productivity under field conditions in *Arabidopsis thaliana*. Franks and Beerling (2009) demonstrated that changes toward increased *D* coupled with reduced *S* could maintain or improve the total stomatal area (caused by increased *D*) but could also provide a shorter diffusion path (caused by the smaller stomatal depth), potentially leading to improved maximum stomatal conductance and photosynthesis. In this study, we identified five accessions, including CX28, CX35, CX182, CX276, and CX343 that carry high-*D* haplotypes of *LOC_Os01g66120* at *qD_*ada*_1* and *OsSPCH2* at *qD_*ada*_2.1* and *qD_*aba*_2.1* (Supplementary Table S5), and three accessions CX230, CX341, and CX362 that carry small-*S* haplotypes of *LOC_Os02g34320* at *qS_*ada*_2.2*, *LOC_Os07g43530* and *OsFLP* at *qS_*aba*_7.1* (Supplementary Table S5). Although these candidates are not causal genes, the SNPs in the gene sequences are suitable for marker-assisted selection (MAS) because of the high degree of linkage disequilibrium between them. Thus, after converting these linked SNPs into Kompetitive Allele Specific PCR (KASP) markers, MAS could be carried out to improve stomatal conductance, thus probably increasing photosynthesis by introgressing the high-*D* alleles (haplotypes) of *LOC_Os01g66120* and *OsSPCH2*, or pyramiding the high-*D* alleles of the above two genes, and the small-*S* alleles of *LOC_Os02g34320*, *LOC_Os07g43530* and *OsFLP* into low-stomatal density varieties.

Rice plants with fewer stomata are better able to maintain stomatal conductance and survive longer than control plants under drought and high temperature (40°C) (Caine et al., 2019). Low-stomatal density rice have achieved equivalent or even increased grain yields, despite a reduced rate of photosynthesis in some stress conditions (Caine et al., 2019). Thus, one strategy for breeding drought tolerant varieties with fewer stomata could be achieved by introgressing the low-*D* alleles of *LOC_Os01g66120* at *qD_*ada*_1* and *OsSPCH2* at *qD_*ada*_2.1* and *qD_*aba*_2.1* into high-stomatal density varieties by MAS. We identified three accessions, including IRIS 313-10211, CX88, and CX123, which carry low-*D* haplotypes of *LOC_Os01g66120* and *OsSPCH2* (Supplementary Table S6). Therefore, these three accessions could be used as donor parents in rice breeding for drought tolerance by MAS.

## Conclusion

The 451 accessions showed wide variations for the eight stomata-related traits. GWAS identified 64 QTLs for the eight traits, 12 of which were consistently detected in nine chromosome regions in the two environments, and 12 QTL regions controlling the same stomata-related traits were simultaneously detected on adaxial and abaxial leaf surfaces in the same environment. A total of 64 candidate genes for the nine consistent QTL regions were identified and the most likely candidate genes for five loci (*qD_*ada*_1*, *qD_*ada*_2.1*/*qD_*aba*_2.1*, *qS_*aba*_7.1*, *qS_*ada*_2.2*, and *qW_*ada*_7*/*qW*_aba_*7*) were inferred based on haplotype analysis and functional annotation. These results will enrich our knowledge of the genetic relationships among stomata-related traits in rice and will provide valuable information to improve rice photosynthesis and stress tolerance by deploying favorable alleles of stomata-related traits by MAS.

## Data Availability Statement

The datasets presented in this study can be found in online repositories. The names of the repository/repositories and accession number(s) can be found in the article/Supplementary Material.

## Author Contributions

YW and JX designed and supervised the research. HC, LZ, KS, KJ, CS, and KC performed the experiments. HC, LZ, and SW analyzed data. YW and JX wrote the manuscript. XZ edited the manuscript. All authors read and approved the manuscript.

## Conflict of Interest

The authors declare that the research was conducted in the absence of any commercial or financial relationships that could be construed as a potential conflict of interest.

## Footnotes

1 https://rapdb.dna.affrc.go.jp/

2 https://ricexpro.dna.affrc.go.jp/

3 http://bbi.irri.org/products
