# Supplementary material for: Genetic Bases of the Stomata-Related Traits Revealed by a Genome-Wide Association Analysis in Rice (Oryza sativa L.)
Source: Front Genet. 2020 Jun 9;11:611. doi: 10.3389/fgene.2020.00611 (PMC7296080; doi:10.3389/fgene.2020.00611)
Supplement: Supplementary file 2 [file Table_2.DOCX]

**TABLE S2** | Variance components and heritability estimated by multiple-site analysis

| Trait | *V*_GEI_ | *V*_G_ | Rep (Env) | *V*_E_ | *V*_e_ | *h*^2^ |
| --- | --- | --- | --- | --- | --- | --- |
| *D*_ada_ | 3017.66 | 4209.41 | 1.74 | 349.86 | 1635.58 | 0.69 |
| *D*_aba_ | 6896.78 | 8063.12 | 0.00 | 1576.71 | 2216.39 | 0.67 |
| *L*_ada_ | 0.76 | 0.67 | 0.00 | 0.02 | 3.02 | 0.41 |
| *L*_aba_ | 0.78 | 0.81 | 0.00 | 0.08 | 0.66 | 0.58 |
| *W*_ada_ | 0.30 | 0.31 | 0.00 | 0.11 | 0.42 | 0.53 |
| *W*_aba_ | 0.31 | 0.39 | 0.00 | 0.14 | 0.24 | 0.65 |
| *S*_ada_ | 164.29 | 124.22 | 0.08 | 23.88 | 76.36 | 0.55 |
| *S*_aba_ | 160.10 | 189.75 | 0.03 | 55.93 | 71.59 | 0.66 |

*D*_ada_, stomatal density on adaxial surface; *D*_aba_, stomatal density on abaxial surface; *L*_ada_, guard cell length on adaxial surface; *L*_aba_, guard cell length on abaxial surface; *W*_ada_, guard cell width on adaxial surface; *W*_aba_, guard cell width on abaxial surface; *S*_ada_, stomatal size on adaxial surface; *S*_aba_, stomatal size on abaxial surface; *V*_E_, environmental variance; *V*_G_, genotypic variance; *V*_GEI_, genotype-by-environment interaction variance; *V*_e_, residual variance; Rep (Env), replication variance within environment; *h*^2^, narrow-sense heritability.
