# Supplementary material for: Genetic Bases of the Stomata-Related Traits Revealed by a Genome-Wide Association Analysis in Rice (Oryza sativa L.)
Source: Front Genet. 2020 Jun 9;11:611. doi: 10.3389/fgene.2020.00611 (PMC7296080; doi:10.3389/fgene.2020.00611)
Supplement: Supplementary file 3 [file Table_3.DOCX]

| **TABLE S3** \| The distribution of single nucleotide polymorphism (SNP) markers on chromosomes | | | |
| --- | --- | --- | --- |
| Chr | Size(Mb) | No. of markers | Average distance (bp) |
| 1 | 43.3 | 359,991 | 120.2 |
| 2 | 35.9 | 279,769 | 128.5 |
| 3 | 36.4 | 252,106 | 144.4 |
| 4 | 35.5 | 247,271 | 143.6 |
| 5 | 29.9 | 196,739 | 152.2 |
| 6 | 31.2 | 248,278 | 125.7 |
| 7 | 29.7 | 220,414 | 134.6 |
| 8 | 28.4 | 246,383 | 115.4 |
| 9 | 22.9 | 168,424 | 136.0 |
| 10 | 23.2 | 213,334 | 108.8 |
| 11 | 29.0 | 263,984 | 109.9 |
| 12 | 27.5 | 240,069 | 114.7 |
| Total/average | 372.9 | 2,936,762 | 127.8 |
