# Supplementary material for: Genetic Bases of the Stomata-Related Traits Revealed by a Genome-Wide Association Analysis in Rice (Oryza sativa L.)
Source: Front Genet. 2020 Jun 9;11:611. doi: 10.3389/fgene.2020.00611 (PMC7296080; doi:10.3389/fgene.2020.00611)
Supplement: Supplementary file 7 [file Data_Sheet_1.PDF]

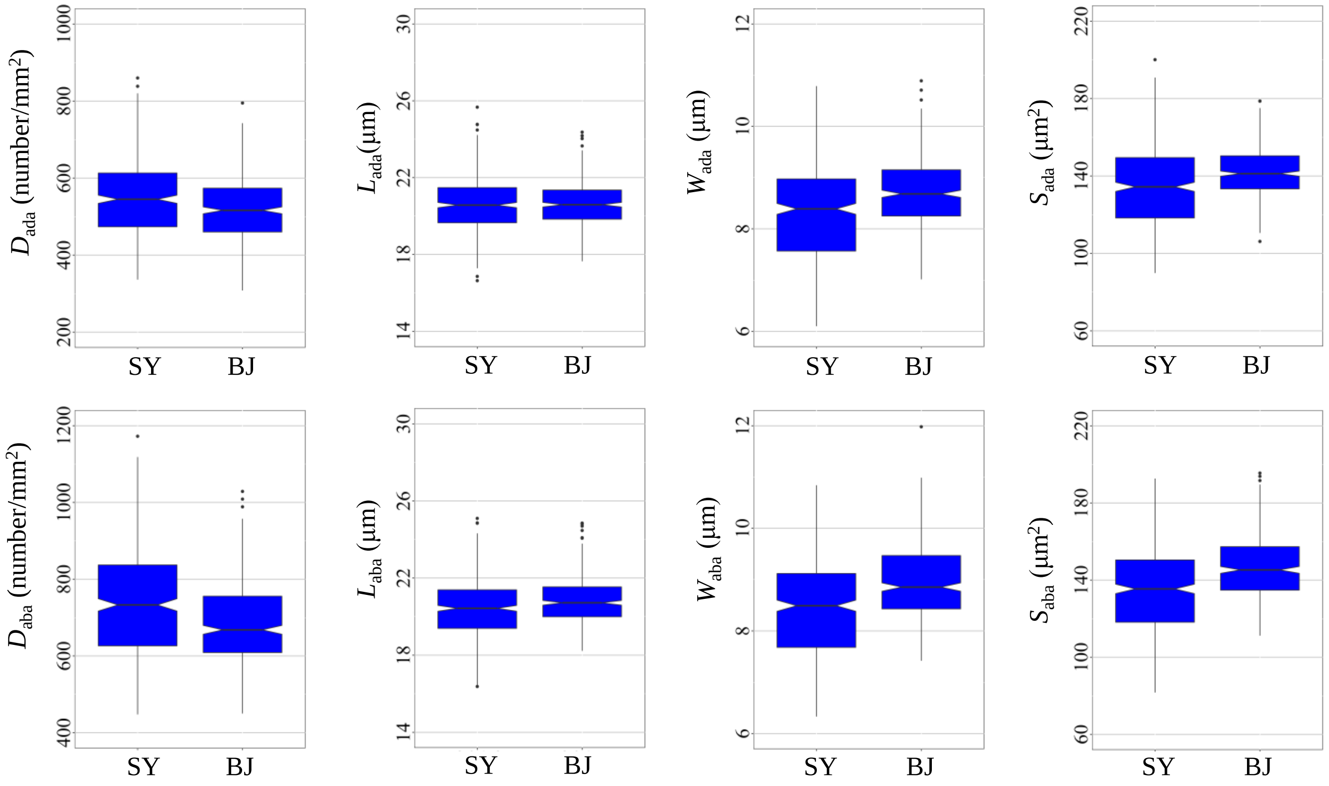


**FIGURE S1** | Box plots of eight stomata-related traits in Sanya (SY) and Beijing (BJ) in 451 accessions. $D_{ada}$, stomatal density on adaxial surface; $D_{aba}$, stomatal density on abaxial surface; $L_{ada}$, guard cell length on adaxial surface; $L_{aba}$, guard cell length on abaxial surface; $W_{ada}$, guard cell width on adaxial surface; $W_{aba}$, guard cell width on abaxial surface; $S_{ada}$, stomatal size on adaxial surface; $S_{aba}$, stomatal size on abaxial surface.
