# Supplementary material for: Genetic Bases of the Stomata-Related Traits Revealed by a Genome-Wide Association Analysis in Rice (Oryza sativa L.)
Source: Front Genet. 2020 Jun 9;11:611. doi: 10.3389/fgene.2020.00611 (PMC7296080; doi:10.3389/fgene.2020.00611)
Supplement: Supplementary file 8 [file Data_Sheet_2.PDF]

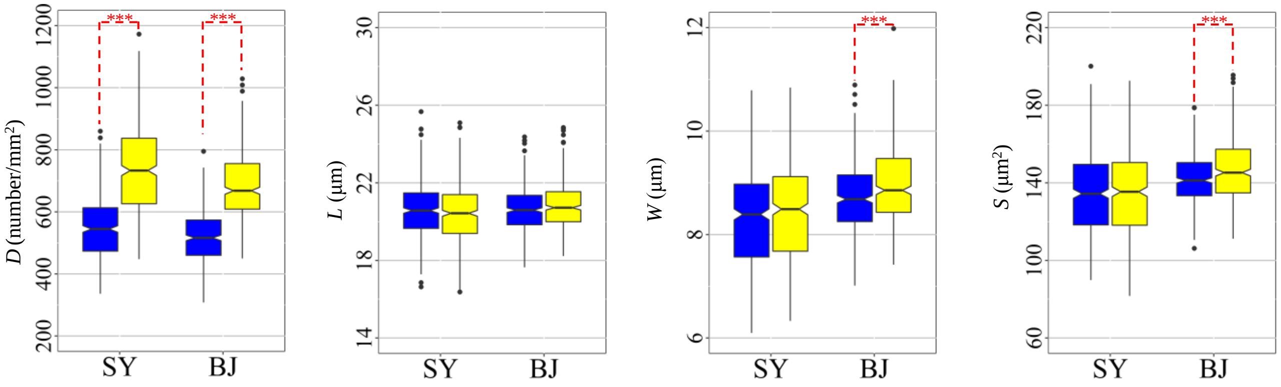

FIGURE S2 | Box plots of stomata-related traits on adaxial and abaxial leaf surface in Sanya (SY) and Beijing (BJ) in 451 accessions. Blue and yellow colors indicate values on adaxial and abaxial leaf surface, respectively. The *** indicate significant correlations at $P < 0.001$. *D*, stomatal density; *L*, guard cell length; *W*, guard cell width; *S*, stomatal size
