# Supplementary material for: Genetic Bases of the Stomata-Related Traits Revealed by a Genome-Wide Association Analysis in Rice (Oryza sativa L.)
Source: Front Genet. 2020 Jun 9;11:611. doi: 10.3389/fgene.2020.00611 (PMC7296080; doi:10.3389/fgene.2020.00611)
Supplement: Supplementary file 9 [file Data_Sheet_3.PDF]

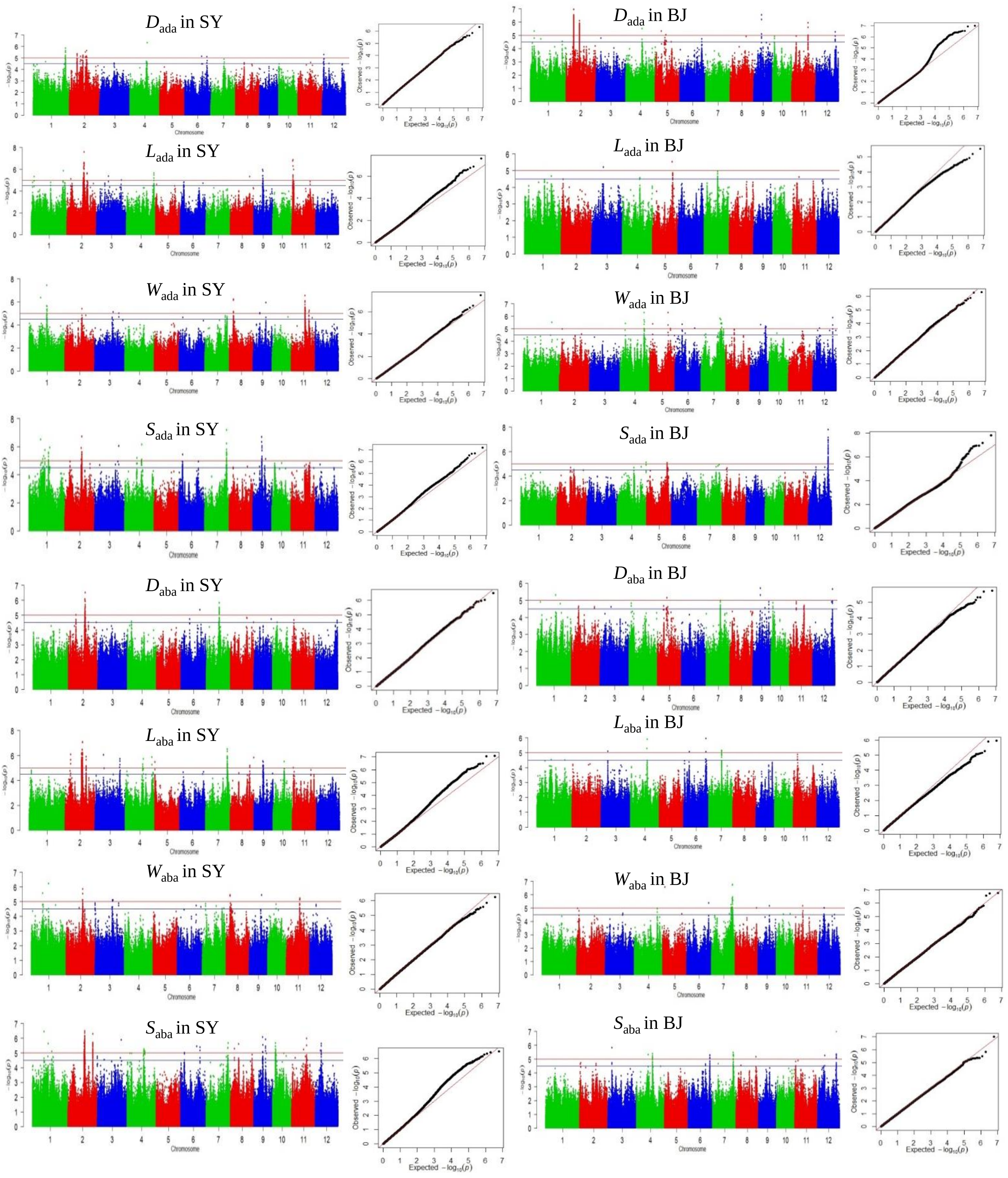

FIGURE S3 | Genome-wide association results for eight stomata-related traits. Manhattan plots (left) and quantile-quantile plots (right) associated with D_{ada}, L_{ada}, W_{ada}, S_{ada}, D_{aba}, L_{aba}, W_{aba}, and S_{aba} in 451 accessions in Sanya (SY) and Beijing (BJ). For the Manhattan plots, -log10 *P*-values from a genome-wide scan were plotted against the position of the SNPs on each of 12 chromosomes and the horizontal red and blue lines show the suggestive threshold $P = 1.0 \times 10^{-5}$, $P = 1.0 \times 10^{-4.5}$, respectively. For the quantile-quantile plots, the horizontal axes indicate the -$\log_{10}$-transformed expected *P* values, and the vertical axes indicate the -$\log_{10}$-transformed observed *P*-values.
